# Emergency department ultrasonography guided long-axis antecubital intravenous cannulation: How to do it

**DOI:** 10.1186/2036-7902-4-3

**Published:** 2012-04-16

**Authors:** David C Riley, Steven Garcia

**Affiliations:** 1 Emergency Medicine Department, Columbia University Medical Center, New York, NY

**Keywords:** ultrasound, ultrasonography, antecubital brachial vein, long-axis, vein cannulation.

## Abstract

An 85-year-old woman with a past medical history of severe peripheral vascular disease and right below knee amputation presented to the emergency department with a 1-day history of non-positional dizziness and weakness. The patient required intravenous access to work up her dizziness and weakness. The patient had multiple failed blind ED peripheral IV attempts performed in the past. Emergency department bedside ultrasonography with a high frequency linear array vascular probe was used to guide antecubital brachial vein cannulation on the first attempt using the long-axis approach.

## Background

Emergency department patients who have failed peripheral IV access attempts often need intravenous fluids, blood, and medications necessitating the need for ultrasound guided peripheral vein catheterization [1-16]. One method for obtaining antecubital venous access in the emergency department is by blindly cannulating the antecubital vein using landmark anatomy and palpation to guide proper position. Each needle pass through the skin and into the patient carries additional risk of complications. Antecubital venous access procedures can be complicated by brachial artery punctures, hematoma formation, brachial nerve injury, severe arm pain post procedure, and catheter malposition [4,5,7,11]. Chinnock reported acute antecubital line complications of antecubital artery puncture in 4% of cases, arm numbness in 3% of cases, and severe arm pain in 7% of cases [4]. In addition, Keyes reported the complications of brachial artery puncture in 2% of cases, and that the IV catheter fell out or was infiltrated within 1 h of placement occur in 8% of emergency department patients with antecubital lines [7]. Multiple catheterization attempts can also lead to upper extremity deep venous thrombosis and increase the blood hemolysis rates [6,8]. Blind cannulation of the antecubital vein increases the rate of complications [4,5,7,11].

The ability to catheterize the antecubital vein on the first attempt is a critical action for an emergency department physician and nurse as it can reduce the time the patient has to wait for important IV fluids, medicines, and blood; and it may reduce both the acute and subacute complications resulting from multiple cannulation attempts. Several investigators have shown that the success rate of first attempt antecubital vein catheterization using the ultrasound guided short-axis approach to the vessel technique is between 43% and 73% [4,5,7,11,15]. Presented is a case of first attempt cannulation success with an ultrasound guided long-axis approach to the antecubital brachial vein (see video clips in Additional file 1).

Ultrasonography can detect anatomic variations, valves, and exact vessel location prior to cannulation. An antecubital vein with preexisting thrombus can also be avoided, and an antecubital vein with atypical anatomy can be identified, and an appropriate ultrasound guided plan for cannulation can be made [4,5,7,11,15]. Their anatomic detail can be better visualized with the long-axis evaluation of the antecubital vessel.

Making the antecubital vein as large as possible increases the chances of cannulating the vessel on the first attempt. Two tourniquets were placed around the patient's upper arm as he isometrically squeezed his fist. Witting has shown that patients with antecubital veins measured less than 4 mm (they did not specify external vs. internal diameter vein measurements) had a 61% overall ultrasound guided antecubital catheterization failure rate [15]. Although Blaivas et al. have shown that novice ultrasound users were able to obtain vascular access faster using the short-axis approach in a vascular phantom model [2], Sierzenski et al. and Stone et al. have shown in vascular phantom models that novice ultrasound users had improved accuracy of identifying the needle tip with a long-axis ultrasound transducer orientation [13,16]. The ability to visualize the needle tip with the long-axis view may be critical for antecubital vein cannulation on the first attempt.

The United States Agency for Healthcare Research and Quality has endorsed the use of ultrasound to guide the placement of central venous catheters to lessen the frequency of complications [12]. There is a critical need to determine if ultrasound used real time for long-axis evaluation of the needle entering the antecubital vessel can result in more successful first attempt antecubital vein cannulations with fewer complications compared to the ultrasound guided short-axis method where the needle is more difficult to visualize entering the antecubital vein [6,7,11].

## Case presentation

An 85-year-old woman with a past medical history of severe peripheral vascular disease and right below knee amputation presented to the emergency department with a 1-day history of non-positional dizziness and weakness. She denied any headache, neck pain, chest pain, trauma, or fever. Her ED vital signs were normal, and her ECG showed normal sinus rhythm with a heart rate of 91 bpm. Her physical examination was normal including a right below knee stump that was clean and dry. An MRI/MRA of the head and neck showed no new changes. The patient required intravenous access to work up her dizziness and weakness. The patient had multiple failed blind ED peripheral IV attempts performed in the past.

**Figure 1:**
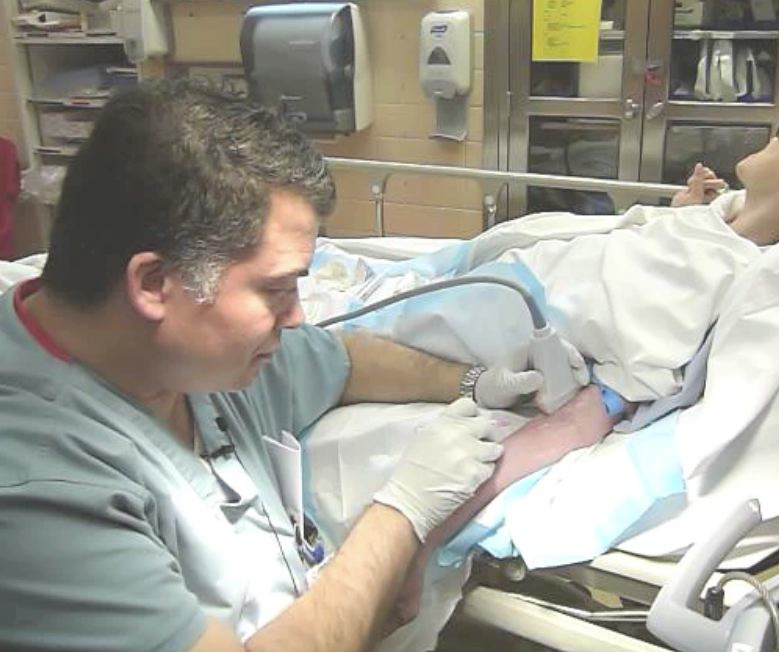
**Dr, Riley performing an ultrasound guided long-axis antecubital intravenous line placement**.

**Figure 2:**
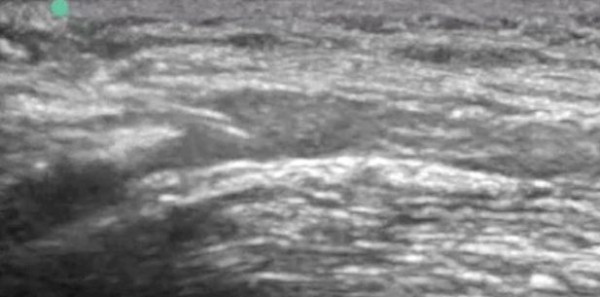
**Needle tip visualized long-axis inside the antecubital vein**.

**Figure 3:**
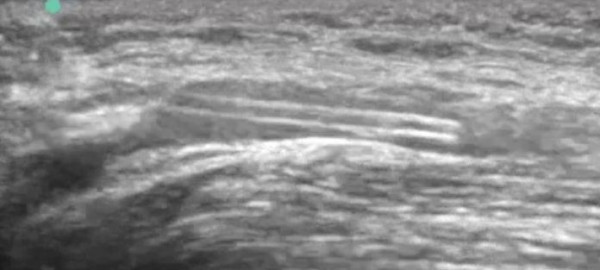
**Catheter visualized long-axis inside the antecubital vein**.

An ED bedside ultrasound guided antecubital brachial vein was cannulated on the first attempt using the long-axis approach (see video clips in Additional files 1, 2, and 3.). After applying an elastic tourniquet to the patients non-dominant left arm and applying ultrasound gel, a high frequency linear array probe was used to identify an antecubital vein that was the largest in diameter, at least 3 mm or greater in diameter and a vein as close as possible to the skin surface. An augmentation procedure was performed by squeezing the patient's forearm while sampling the vessel with color Doppler and pulse Doppler. The augmentation procedure with increased Doppler flow verified the selected vessel was a vein, since an arterial vessel would produce a pulsatile Doppler flow pattern (see video clip in Additional file 2.). The skin was prepped with alcohol swabs, the vein verified with augmentation again, and the probe, now covered with a thin sterile plastic film dressing, was placed in the nine o'clock position to view the antecubital brachial vein in short-axis, then the ultrasound probe was turned counter clockwise to the six o'clock position to position the vein in the long-axis position (Figure 1). The ED physician held the vascular ultrasound probe while resting on the patient's bed to provide stability. A 20-gauge 2-in. catheter was placed under the long-axis of the probe and vein so that direct visualization of the needle tip and catheter inside the vessel could occur (Figures 2 and 3). The catheter was flushed with saline and secured.

## Conclusions

Emergency department bedside ultrasonography with a high frequency linear array vascular probe can be used to guide antecubital brachial vein cannulation on the first attempt using the long-axis approach.

## Consent

Written informed consent was obtained from the patient for publication of this case report and any accompanying images. A copy of the written consent is available for review by the Editor-in-Chief of this journal.

## Competing interests

The authors declare that they have no competing interests.

## Authors' contributions

DR drafted the manuscript, and SG participated in and helped develop the video demonstration. All authors read and approved the final manuscript.

## Authors' Information

DR is the director of Emergency Ultrasonography and Ultrasound Research at the Emergency Medicine Department, Columbia University Medical Center, New York, NY.

SG is an emergency medicine resident in the New York Presbyterian, Columbia/Cornell training program, New York, NY.

## Supplementary Material

Additional file 1 **Emergency department ultrasonography guided long-axis antecubital intravenous cannulation-part 1**. Introduction, equipment needed, and contraindications for placing an ultrasound guided antecubital intravenous catheter. Click here for file

Additional file 2 **Emergency department ultrasonography guided long-axis antecubital intravenous cannulation-part 2**. Demonstration of initial short-axis probe positioning and transitioning to a long-axis approach to the antecubital vein. Augmentation technique demonstrated to confirm that the blood vessel is a vein. Click here for file

Additional file 3 **Emergency department ultrasonography guided long-axis antecubital intravenous cannulation-part 3**. Demonstration of placing the needle catheter with ultrasound long-axis guidance into the antecubital vein with good blood flow. Click here for file
